# Metaheuristics in the Optimization of Cryptographic Boolean Functions

**DOI:** 10.3390/e22091052

**Published:** 2020-09-21

**Authors:** Isaac López-López, Guillermo Sosa-Gómez, Carlos Segura, Diego Oliva, Omar Rojas

**Affiliations:** 1 Centro de Investigación en Matemáticas A.C. (CIMAT). Área de Computación, Jalisco S/N, Col. Valenciana, Guanajuato 36023, Mexico; isaac.lopez@cimat.mx (I.L.-L.); carlos.segura@cimat.mx (C.S.); 2 Facultad de Ciencias Económicas y Empresariales, Universidad Panamericana, Álvaro del Portillo 49, Zapopan, Jalisco 45010, Mexico; orojas@up.edu.mx; 3 IN3-Computer Science Department, Universitat Oberta de Catalunya, 08860 Castelldefels, Spain; diego.oliva@cucei.udg.mx; 4 Depto. de Ciencias Computacionales, Universidad de Guadalajara, CUCEI, Av. Revolución 1500, Guadalajara C.P. 44430, Jalisco, Mexico

**Keywords:** boolean function, metaheuristics, nonlinearity, cryptography, hadamard transform, entropy

## Abstract

Generating Boolean Functions (BFs) with high nonlinearity is a complex task that is usually addresses through algebraic constructions. Metaheuristics have also been applied extensively to this task. However, metaheuristics have not been able to attain so good results as the algebraic techniques. This paper proposes a novel diversity-aware metaheuristic that is able to excel. This proposal includes the design of a novel cost function that combines several information from the Walsh Hadamard Transform (WHT) and a replacement strategy that promotes a gradual change from exploration to exploitation as well as the formation of clusters of solutions with the aim of allowing intensification steps at each iteration. The combination of a high entropy in the population and a lower entropy inside clusters allows a proper balance between exploration and exploitation. This is the first memetic algorithm that is able to generate 10-variable BFs of similar quality than algebraic methods. Experimental results and comparisons provide evidence of the high performance of the proposed optimization mechanism for the generation of high quality BFs.

## 1. Introduction

In several cryptographic systems and in the use of noisy channels, Boolean Functions (BFs) play an important role. Specifically, BFs are used in the internal operation of many cryptographic algorithms, and it is known that in order to avoid the success of cryptanalysis on such systems, increasing the degree of nonlinearity of BFs is utterly important. The research in Cryptographic Boolean Functions (CBFs) has increased significantly in the last few decades. Particularly, the cryptographic community has been widely working in the problem of generating BFs with good cryptographic properties. One of the first authors to study such topic showed the important implication of the feature known as *correlation immunity* using algebraic procedures [1]. CBFs are currently used in symmetric-key cryptography both in block and stream ciphers [2]. In both of these types of ciphers, the only nonlinear elements are usually the BFs applied in the stream ciphers and the vectorial BFs (better known as substitution boxes or s-boxes) in the block ciphers. Without the use of BFs in stream ciphers, or s-boxes in block ciphers, it would be trivial to break the cryptographic systems. As a result, designing proper BFs is a crucial step.

Several efforts have been devoted to finding proper CBFs to avoid cryptanalytic attacks [3]. Thus, cryptographers study the desired properties for CBFs. In particular, some desired features are high nonlinearity, balancedness, algebraic degree and low autocorrelation. Attaining balanced BFs with high nonlinearity is one of the most challenging tasks [3] and it is particularly important because they hinder the application of linear and differential cryptanalysis.

Currently, many ways to generate CBFs with proper features have been designed. The three main approaches that are currently used are [4] algebraic constructions, random generation and metaheuristic constructions. Algebraic constructions use mathematical procedures based on algebraic properties to create BFs with good cryptographic properties. Random generation is easy and fast, but the resulting BFs usually have suboptimal properties. Finally, metaheuristic constructions are heuristic techniques that have attained quite promising BFs and are easier to design than algebraic methods. However, up to now, metaheuristic techniques have not been able to attain so good results as the algebraic techniques. For instance, attending to the nonlinearity for balanced CBFs, metaheuristics attain CBFs with lower nonlinearity than the best-known CBFs that have been generated with algebraic constructions. Note that in practice, it is first necessary to have methods that allow obtaining BFs with high nonlinearity and then, as is the trend in the literature, compromise nonlinearity to increase resistance to DPA attacks, Side Channel, and so forth [5]. Alternatively, the different features might be tackled simultaneously with multi-objective optimizers (MO). In fact, in the related literature there exist different implementations following this principle [6]. However, in our work we focus only on the nonlinearity so single-objective optimizers are applied.

Regarding the generation of highly nonlinear CBFs, one of the main difficulties is that the search space $F_{n}$ is immensely large: $|F_{n}|=2^{2^{n}}$. In fact, for problems with more than five variables, it is not possible to do an exhaustive search. Furthermore, as *n* increases, not only the search space grows, but also the computational cost of calculating the various important properties increases. Specifically the calculus of the nonlinearity is based on the usage of the Walsh Hadamard Transform (WHT), so there are more BFs and estimating the quality of each BF is more costly. This article focuses on the *n*-variable BF problem, with n=8,10. The main efforts are concentrated in further improving the highest nonlinearity achievable by metaheuristics for these *n*-variable BF problems. Note that some researchers consider that balanced BFs for 8 variables with nonlinearity equal to 118 do not exist, so the best-known BF might already be known [7]. This function has been searched for at least since 30 years ago but its existence has not been proved [8]. A similar situation appears for the case of 10-variable BFs with nonlinearity equal to 494 [7]. In any case, the main purpose of this research is to reduce the gap between algebraic methods and metaheuristic approaches [8]. Notice that some novel general purpose strategies are designed so the advances put forth in this paper might be applied in additional fields of optimization.

Diversity-aware population based metaheuristics have provided important benefits in recent years. Particularly, they have been able to generate new best-known solutions in several combinatorial optimization problem [9]. This paper studies the hypothesis that the state of the art in the generation of balanced CBFs with high nonlinearity can be improved further with proper diversity-aware memetic algorithms. To the best of our knowledge, our proposal is the first population-based approach that considers the diversity in an explicit way in order to generate balanced CBFs with high nonlinearity. The main novelty appears in the replacement phase, where concepts related to clustering and diversity are applied. Additionally, two new cost functions that guide the search towards proper regions are devised. Regarding the diversity management, two strategies that involve different ways of controlling the diversity are applied. The first one, which was successfully devised for the Graph Partitioning Problem [10], enforces a large contribution to diversity for every member of the population, whereas the second one, which is a novel proposal, considers the creation of clusters, meaning that some close members are maintained in the population. The lower entropy induced by the formation of clusters [11] allows the promotion of a larger degree of intensification, which is a key to success. Finally, a novel population initialization method is also proposed which considers some basic algebraic constructions with the aim of speeding up the attainment of high quality BFs. Concerning the results, we remark that our proposals reduce the gap between algebraic and heuristic constructions. In fact, with the methods proposed in this work, the results obtained by algebraic constructions for 8 and 10 variables have been matched.

This work is organized as follows. Section 2 formally defines our problem and introduces the main required concepts. Section 3 provides a review of metaheuristics applied to the generation of CBFs as well as other optimizers that guided our design. Trajectory-based and population-based proposals are described and analyzed is Section 4 and Section 5, respectively. Parameterization studies and comparisons against the best-known solutions are included. Finally, conclusions and some lines of future work are given in Section 6.

## 2. Problem Statement: Formal Definitions

The problem addressed in this paper is the generation of balanced CBFs with high nonlinearity. This section formally defines the concepts and algorithms required to fully understand the problem.

### 2.1. Boolean Functions and Representations

The set {0,1} is the most often endowed with the structure of a field (denoted by $F_{2}$) and the set $F_{2}^{n}$ of all binary vectors of length *n* is viewed as an $F_{2}$ vector space. The null vector of $F_{2}^{n}$ is 0. $F_{2}^{n}$ is endowed with a field structure to form the well-known Galois Field $\mathrm{GF}(2^{n})$ [12]. Given these basic definitions, a *n*-variable BF is a function

(1) $$f:F_{2}^{n}\to F_{2}.$$

It is usually written as $f\left(x\right)=f\left(x_{1},x_{2},\ldots ,x_{n}\right)$, where x is the shorthand writing of vector $\left(x_{1},x_{2},\ldots ,x_{n}\right)$. Note that when considering *n* variables, there are $|F_{n}|=2^{2^{n}}$ different Boolean functions.

BFs can be represented in several ways. Some of the most typical are the algebraic forms, the *binary truth table* and the *polarity truth table*. The *binary truth table* is the lexicographically ordered *vector* of all outputs of the BF *f*. The binary truth table has length $2^{n}$. The lexicographical order of the binary vectors x follows a particular order when going through all the possible values in $F_{2}^{n}$. Let $k_{x}$ denote the integer representation of $x=(x_{1},x_{2},\ldots ,x_{n})$, that is,

(2) $$k_{x}=to\_int\left(x\right)=\sum_{i=1}^{n}x_{i}2^{n-i}.$$

We can see the function to_int as the function that maps a vector x from $F_{2}^{n}$ to an integer $k_{x}\in N_{n}=\left\{0,\cdots ,2^{n}-1\right\}$. When the integer $k_{x}$ takes all the values from 0 incrementally to $2^{n}-1$, then the corresponding binary vector x goes through all the elements in $F_{2}^{n}$. This order is the lexicographical one. Linked to this representation is the *polarity truth table* or *polar form*. Let *f* denote a BF, then $\hat{f}$ is used to define the *polarity truth table*. In this case $\hat{f}\left(x\right)\in \;\left\{-1,1\right\}$ and each element of $\hat{f}$ is obtained as $\hat{f}\left(x\right)={\left(-1\right)}^{f(x)}$.

*Affine BFs* on $F_{n}$ are those that can be expressed as $L_{w,c}$:

(3) $$L_{w,c}\left(x\right)=w\cdot x⊕c=w_{1}x_{1}⊕\cdots ⊕w_{n}x_{n}⊕c,$$

where $w\in F_{2}^{n}$ and $c\in F_{2}$. If c=0, then $L_{w,0}$ ($L_{w}$) is a linear BF. The sets of *n*-variable affine and linear BFs are denoted by $A_{n}$ and $L_{n}$, respectively. They are important because they are involved in the calculus of the nonlinearity. The number of *n*-variable affine and linear BFs are

(4) $$\begin{array}{cc} |A_{n}| & =2^{n+1}, \end{array}$$

(5) $$\begin{array}{cc} |L_{n}| & =2^{n}. \end{array}$$

Additional important concepts are the *Hamming Weight* and the *Hamming Distance*. The Hamming Weight of a BF *f* is defined as the numbers of $1^{\prime }s$ in its truth table and is denoted by $w_{H}\left(f\right)$:

(6) $$w_{H}\left(f\right)=\sum_{x\in F_{2}^{n}}f\left(x\right).$$

This definition is also true for vectors $x\in F_{2}^{n}$.

Let f(x), g(x) be two *n*-variable BFs, the *Hamming distance* between f(x) and g(x), denoted by $d_{H}\left(f,g\right)$, is the number of coordinates with different values in their truth tables. It can be written as

(7) $$d_{H}\left(f,g\right)=w_{H}\left(f⊕g\right)=\sum_{x\in F_{2}^{n}}f\left(x\right)⊕g\left(x\right).$$

### 2.2. Walsh Hadamard Transform

The *Walsh Hadamard Transform* (WHT) can be seen as another kind of *n*-variable BF representation that is useful to calculate relevant cryptographic properties of BFs, such as the nonlinearity. Let *f* be a *n*-variable BF, the WHT of *f* is the function $W_{n}:F_{2}^{n}\to Z$ defined by

(8) $$W_{n}\left(f\right)\left(w\right)=\sum_{x\in F_{2}^{n}}{\left(-1\right)}^{f\left(x\right)⊕L_{w}\left(x\right)},\;w\in F_{2}^{n}.$$

WHT can also be calculated by using the polarity truth tables of *f* and $L_{w}$

(9) $$W_{n}\left(\hat{f}\right)\left(w\right)=\sum_{x\in F_{2}^{n}}\hat{f}\left(x\right){\hat{L}}_{w}\left(x\right),\;w\in F_{2}^{n}.$$

The WHT of *f* measures the correlation between *f* and each $L_{w}$. According to Millan [3], the correlation with $L_{w}$ is given by

(10) $$c\left(f,L_{w}\right)=\frac{|W_{n}\left(f\right)\left(w\right)|}{2^{n}}.$$

The correlation is a real number $0\leq c(f,L_{w})\leq 1$, that represents the degree of similarity between *f* and $L_{w}$. A correlation value equal to zero indicates that *f* and $L_{w}$ are completely uncorrelated; a value equal to 1 means a perfect correlation between *f* and $L_{w}$.

Note that direct calculation of $W_{n}$ would require about $2^{2n}$ operations. This is because there exists $2^{n}$ linear functions, and computing the correlation with each linear function requires $2^{n}$ operations. Fortunately there is a faster way to obtain $W_{n}$, the Fast Walsh Hadamard Transform (FWHT), which is a discrete version of the so-called Fast Fourier Transform (FFT). Since our optimizer requires the calculation of the WHT, the FWHT is used.

#### Parseval’s Equation

The values in the WHT of *f* are constrained by a square sum relationship which implicitly limits the magnitude and frequency of those values, this is known as Parseval’s Equation (11) [13]:

(11) $$\sum_{w\in F_{2}^{n}}{\left(W_{n}\left(f\right)\left(w\right)\right)}^{2}=2^{2n}.$$

This value is constant for all *n*-variable BFs, that is, the values in the WHT of every BF must satisfy Parseval’s equation. However, a WHT of a function can satisfy Parseval’s equation and not necessarily be Boolean. For this reason, even if the WHT is required, optimization methods usually operate with encoding based on truth tables.

### 2.3. Cryptographic Properties of Boolean Functions and Special Functions

Boolean functions used in stream ciphers must have some required properties with the aim of making ciphers secure against some attacks. Some of the most important cryptographic properties of BFs are the algebraic degree, the balancedness, the nonlinearity and the auto correlation. In this work we consider the balancedness and nonlinearity.

The balancedness is one of the most basic of all cryptographic properties desired to be exhibited by BFs. *f* is said to be balanced if $w_{H}\left(f\right)=2^{n-1}$. In terms of its WHT, a BF *f* is balanced if and only if

(12) $$W_{n}\left(f\right)\left(0\right)=0.$$

The set of *n*-variable balanced BFs is denoted as $B_{n}$ and its size is

(13) |Bn|=2n2n−1.

It is important to remark that we use this search space along this paper, that is, our optimizer does not generate unbalanced BFs.

The nonlinearity $N_{n}\left(f\right)$ of *f* is calculated using the maximum absolute value of the WHT and represents the minimum Hamming distance between *f* and the affine BFs set $A_{n}$, that is,

(14) $$N_{n}\left(f\right)=\min_{A_{w,c}\in A_{n}}d_{H}\left(f,A_{w,c}\right).$$

Mathematically, the relationship between the nonlinearity of a *n*-variable BF *f* and the WHT of *f* is given by the following equation:

(15) $$N_{n}\left(f\right)=\frac{1}{2}\left(2^{n}-\max_{w\in F_{2}^{n}}\left|W_{n}\left(f\right)\left(w\right)\right|\right),$$

where $\max_{w\in F_{2}^{n}}\left|W_{n}\left(f\right)\left(w\right)\right|$ represents the maximum absolute value in the WHT. By Parseval’s equation, we can obtain an upper bound of nonlinearity in the general case (when *n* is even), that is,

(16) $$N_{n}\left(f\right)\leq 2^{n-1}-2^{n/2-1}.$$

Bent BFs are a very special class of BFs. A BF f(x) is bent if and only if

(17) $$|W_{n}\left(f\right)\left(w\right)|=2^{n/2},\;\forall w\in F_{2}^{n}.$$

Bent BFs are not balanced and they are not applied usually in cryptosystems. It can be noticed that bent BFs only exist when *n* is even. While they are not applied directly in our proposal, they inspired one of the methods applied to initialize the population in this paper.

## 3. Literature Review

This section reviews related papers on the application of metaheuristics to the generation of CBFs and some recent advances in population-based metaheuristics that guided the design of our proposals.

At least since two decades ago, the firsts researchers started to study the design of CBFs from the metaheuristics point of view. The first *Genetic Algorithm* (GA) that tries to maximize the nonlinearity of BFs was proposed in Reference [14]. The importance of including an intensification stage was shown by applying a smart version of a hill climbing method [3]. The superiority of memetic algorithms against pure GAs was shown. Subsequently, a novel crossover operator to find BFs with high nonlinearity was proposed [15]. This operator combines two balanced CBFs and produces a single balanced CBF.

More recently, it was noted that an important step in the application of metaheuristics for the generation of CBFs is the design of proper fitness or cost functions [16]. This last study was developed by applying *Simulated Annealing*. Subsequently, the proposal was extended by designing a more sophisticated search technique of Simulated Annealing called “vanilla” [17] with the aim of obtaining CBFs with some additional properties such as resilience. Authors note that the huge search space hinders the attainment of better results. Obviously, this is not a drawback specific to this technique. In order to partially avoid this drawback, some authors consider some of the advances performed with algebraic constructions. Particularly, References [18,19] proposed trajectory-based search methods that operate with *bent BFs*. They randomly adjust the bent BF to convert it on a balanced BF. In this way, they decrease the nonlinearity of a bent BF instead of increasing the nonlinearity of a randomly created BF. First, a bent BF is constructed with the method given in Reference [20]. Then, a trajectory-based heuristic is used to attain a balanced BF. These methods show really good results although not as good as more complex algebraic approaches. In fact, at the starting point of our research, these methods were the state-of-the-art in the generation of BFs with high nonlinearity by applying metaheuristics. Given the promising behavior of this method, one of the optimizers put forth in this paper applies a simple algebraic techniques in the generation of the initial population. A different alternative to deal with the so large search space in the case of maximizing the nonlinearity for balanced BFs, is to take into account the symmetries that appear in the fitness landscape when using the bit string representation [21].

Some other recent works [22] include comparisons among different metaheuristics such as GAs, Evolution Strategies (ESs) and Cartesian Genetic Programming (CGP). Among the tested approaches, CGP was the best one to generate 8-variable CBFs with high nonlinearity. In fact, it is the EA with best performance among those that do not apply the concept of bent BFs. Another contribution of this paper is to compare three different fitness functions with the aim of increasing the nonlinearity.

Finally, some notions on the difficulty of generating balanced BFs with high nonlinearity are discussed in Reference [23]. This last study is developed using *Estimation of Distribution Algorithms* (EDAs). They note some undesired properties of using the nonlinearity as fitness function.

Regarding the use of multi-objective optimization algorithms, there are important works including several metaheuristics. For example in Reference [6] the authors propose the use of a MO Genetic Programming algorithm. They used three objective functions that must be maximized: the nonlinearity, algebraic degree, and correlation immunity. In the same context, Non-dominated sorting genetic algorithm II (NSGA-II) was also implemented to construct cryptographically strong Boolean functions [24] by optimizing nonlinearity, resiliency and autocorrelation. Authors remark that the use of MO increased the computational cost.

Recent advances in metaheuristics have shown that in many combinatorial optimization problems, results are advanced further with diversity-aware memetic algorithms [25]. Particularly, relating the diversity maintained in the population to the stopping criterion and elapsed period with the aim of balancing the search from exploration to exploitation as the evolution progresses seems very promising [26]. This principle has not been used in current state-of-the-art metaheuristics for CBFs. Taking into account the difference among the best-known BFs and the ones attained by state-of-the-art metaheuristics the main aim of this paper is to advance further the development of metaheuristic construction techniques. Particularly, the paper focuses on the development of novel memetic algorithms incorporating explicit control of diversity that follow the aforementioned design principle.

## 4. Trajectory-Based Proposals

Since memetic algorithms attain BFs with higher nonlinearity than pure GAs [3], our diversity-aware population-based proposals are memetic approaches, meaning that a trajectory-based strategy is applied to promote intensification. One of our first steps in this research was designing and analyzing different trajectory-based proposals. This section is devoted to fully detail our trajectory-based proposal. In order to fully define our strategy, the representation of solutions, the applied cost function and the details regarding the neighborhood and selection approaches are given.

### 4.1. Representation of Solutions

In this paper each solution S is encoded using a binary representation composed of a vector with $2^{n}$ Boolean decision variables S[k], where *n* is the number of variables of the BF *f* and $k\in N_{n}$. In order to introduce the relationship between the solution S and *f*, let us introduce the analogous function that maps an integer *k* to a binary vector $x\in F_{2}^{n}$, thus, $to\_bin:N\to F_{2}^{n}$, such that

(18) $$x=\left(x_{1},\ldots ,x_{n}\right)=to\_bin\left(k\right)=\left(\left\lfloor \frac{k}{2^{n-1}}\right\rfloor \%2,\ldots ,\left\lfloor \frac{k}{2^{n-n}}\right\rfloor \%2\right).$$

Now, we are ready to introduce the relationship between the solution S and *f*. Each decision variable S[k] is set equal to the corresponding truth table value f(x), where $k=to\_int\left(x\right),x\in F_{2}^{n}$ (or $x=to\_bin\left(k\right),k\in N_{n}$)

(19) f(x)=S[k].

Note that both our trajectory-based and population-based proposals operate with balanced BFs, so $2^{n-1}$ decision variables are set to 0 and $2^{n-1}$ decision variables are set to 1. In order to facilitate some descriptions, let us define the positions sets $P_{0}$ and $P_{1}$ as the set of positions whose values are 0 and 1, respectively. The sets $P_{0}$ and $P_{1}$ have the following properties:

$$|P_{0}\left|=\right|P_{1}|=2^{n-1},\;P_{0}\cap P_{1}=\emptyset ,\;P_{0}\cup P_{1}=N_{n}.$$

Each solution has associated its $P_{0}$, $P_{1}$ sets and an integer vector W of length $2^{n}$ which denotes the WHT of the BF *f*. Analogously to the equality (19) we have that the following holds for a vector W denoting the WHT of the BF *f*

(20) $$W_{n}\left(f\right)\left(w\right)=W\left[k\right]\;s.t\;k\in N_{n},\;w=to\_bin\left(k\right),$$

where W[k] denotes an integer value and $W_{n}\left(f\right)\left(w\right)$ the WHT value associated to the linear function $L_{w}$, which is also an integer.

### 4.2. Neighborhood

In order to define the neighborhood employed in this paper it is necessary to define an operator that transforms a solution S into another one $S^{\prime }$. We call this operator *Bit Swapping* (BS). BS receives a solution S and two positions $p_{0}$ and $p_{1}$ as inputs and returns another solution $S^{\prime }$, such that the decision variables $S[p_{0}]$ and $S[p_{1}]$ are exchanged (or complemented) and $d_{H}\left(S,S^{\prime }\right)=2$, that is,

(21) $$S^{\prime }\left[k\right]=\left\{\begin{array}{cc} 1⊕S[k] & \;\mathrm{if}\;k\in \{p_{0},p_{1}\}\\ \;\;\;S[k] & \;\mathrm{if}\;k\in N_{n}\backslash \left\{p_{0},p_{1}\right\}. \end{array}\right.$$

The Bit Swapping operator is defined as follows

(22) $$BS\left(S,p_{0},p_{1}\right):=swap\left(S\left[p_{0}\right],S\left[p_{1}\right]\right).$$

Employing BS we can construct the full neighborhood for a single solution S as

(23) $$N\left(S\right)=\{BS\left(S,p_{0},p_{1}\right):p_{0}\in S.P_{0}\wedge p_{1}\in S.P_{1}\}.$$

The total amount of bit-swapping that can be performed is the neighborhood size

(24) $$|N\left(S\right)|=|S.P_{0}|\times |S.P_{1}|=2^{n-1}\times 2^{n-1}=2^{2n-2}.$$

### 4.3. Cost Functions

In order to properly measure the performance of the algorithms developed in this paper, it is important to remark that our aim is to generate balanced BFs with high nonlinearity, that is, our objective function is the nonlinearity

(25) $$F_{1}\left(S\right)=\frac{1}{2}\left(2^{n}-\max_{k\in N_{n}}\left|S.W\left[k\right]\right|\right).$$

We can analyze some properties of this function by taking into account that the values in the WHT S.W are constrained by the Parseval’s equation. We know that for a balanced BF all the values in its WHT are multiples of 4. This has as a consequence that the amount of different values that could take $\max_{k\in N_{n}}\left|S.W\left[k\right]\right|$ is really small so the different values of $F_{1}$ are also small. This relationship between $\max_{k\in N_{n}}\left|S.W\left[k\right]\right|$ and $F_{1}$ is clearer if we transform the original objective function (maximize the nonlinearity) into another one (minimize the maximum value in the WHT):

(26) $$F_{2}\left(S\right)=\max_{k\in N_{n}}\left|S.W\left[k\right]\right|.$$

Similarly to other researchers [27], we realized that using the objective function as the fitness function is not suitable to guide the search in EAs and it is necessary to define a new fitness function (in our case a cost function). One of the drawbacks seen easily from the objective function $F_{1}$ or $F_{2}$ is that it just takes a small amount of different values, so a lot of BFs are considered to be of equal value. However, even if they share the same nonlinearity, some of them could be used to easily improve the nonlinearity by just making some modifications, while another ones could be far from better solutions.

The most popular cost function—since it is a cost function it must be minimized—used with metaheuristics is the objective function $F_{2}$, that will be denoted as $C_{1}$ in the following:

(27) $$C_{1}\left(S\right)=F_{2}\left(S\right)=\max_{k\in N_{n}}\left|S.W\left[k\right]\right|.$$

Also, the cost function designed by Clark [27] is widely used,

(28) $$C_{2}\left(S\right)=\sum_{k=0}^{2^{n}-1}{\left||S.W\left[k\right]|-X\right|}^{R}.$$

When using $C_{2}$ every value in the WHT influences the cost function of *S*, rather than just the maximum one as in the cost function $C_{1}$. The authors note “the parameters X and *R* provide freedom to experiment”. Similarly to the authors, we use R=3 and for the X, the expected maximum value for the WHT when it achieves the nonlinearity upper bound.

One of the contributions of this paper is the definition of new cost functions. We propose two new cost functions in order to address the problem properly. In order to explain our cost function, lets denote the absolute values appearing in the WHT as $X_{1},X_{2},\cdots ,X_{\alpha }$, such that

$$X_{1}=0<X_{2}<\cdots <X_{\alpha }=\max_{k\in N_{n}}\left|S.W\left[k\right]\right|.$$

In addition, $\eta_{i}$ is used to denote the amount of times that the $X_{i}$ or $-X_{i}$ value appears in the WHT. Note that $X_{1}=0$, since S.W[0]=0 and $\eta_{1}>0$. Based on this, we propose two new cost functions. In both we form a tuple of values

(29) $$\begin{array}{cc} C_{3}\left(S\right) & =(F_{2}\left(S\right),\xi_{1}\left(S\right)), \end{array}$$

(30) $$\begin{array}{cc} C_{4}\left(S\right) & =(F_{2}\left(S\right),\xi_{2}\left(S\right)), \end{array}$$

where the first component is the objective function $F_{2}$ and the second components $\xi_{1}$, $\xi_{2}$—which are the novelty—help us to discern between solutions with the same nonlinearity. The second members $\xi_{1},\xi_{2}$ of such cost functions are the following:

1. In the second value of $C_{3}$ we try to minimize the maximum absolute value $X_{\alpha }$ and the second maximum absolute value $X_{\alpha -1}$ in the WHT. In order to attain this aim, it takes also into account the number of appearances of these values ($\eta_{\alpha }$ and $\eta_{\alpha -1}$) in the WHT. This is implemented as follows:
  (31) $$\xi_{1}\left(S\right)={\left(\eta_{\alpha }\times X_{\alpha }\right)}^{3}+\left(\eta_{\alpha -1}\times X_{\alpha -1}\right).$$
2. Let $X_{k}$ be a value such that $X_{k-1}\leq X<X_{k}$, with X defined as in Reference [12]. In the second value of the cost function, we try to minimize the appearance of entries with absolute values greater than X assigning larger penalties to larger values:
  (32) $$\xi_{2}\left(S\right)=\sum_{i=k}^{\alpha }{\left(2\times \eta_{i}\times X_{i}\right)}^{i-k+1}.$$

In order to employ the cost functions previously defined, we define the new operators ≺ and ⪯ to identify if a solution $S^{\prime }$ is better than a solution S ($S^{\prime }≺S$) or if a solution $S^{\prime }$ is better or equal than a solution S ($S^{\prime }⪯S$). It used the lexicographic comparison. For example, for $C_{3}$, we have

(33) $$\begin{array}{cc} S^{\prime }≺S & :F_{2}\left(S^{\prime }\right)<F_{2}\left(S\right)\vee \left(F_{2}\left(S^{\prime }\right)=F_{2}\left(S\right)\wedge \xi_{1}\left(S^{\prime }\right)<\xi_{1}\left(S\right)\right), \end{array}$$

(34) $$\begin{array}{cc} S^{\prime }⪯S & :F_{2}\left(S^{\prime }\right)<F_{2}\left(S\right)\vee \left(F_{2}\left(S^{\prime }\right)=F_{2}\left(S\right)\wedge \xi_{1}\left(S^{\prime }\right)\leq \xi_{1}\left(S\right)\right). \end{array}$$

### 4.4. Full Hill Climbing

The most straightforward trajectory-based approach is full hill climbing (FHC) [28]. In hill climbing the neighborhood is completely explored and only improvement movements are accepted. There are several strategies to select among the neighbors that improve the current solution. In stochastic hill climbing, which is the variant that we applied, it is selected randomly. Algorithm 1 illustrates the working operation of FHC. This algorithm makes use of the neighborhood (23). FHC ensures that all the neighbors of a solution S are visited just once. First one decision variable set equal to 0 is selected at random (line 5) and it is swapped with all the decision variables set equal to 1 (line 6). Then if any of the neighbors generated is better than the current solution, this is selected to replace the current solution S (line 9) and both for cycles are restarted. If no better neighbor is found and the neighborhood is fully visited, the solution is a local optimum and the algorithm stops (line 16). Another stopping criterion that might be used is the elapsed time (line 12) returning the best solution found so far.

In order to perform a comparison among the four cost functions previously detailed, they were integrated in FHC and tested for n=8,10 and it was executed 50 times with different seeds. FHC was executed until a local optimum was reached. Table 1 summarizes the results obtained for 50 independent runs. Specifically, for each cost function the minimum, maximum, mean, median and standard deviation of the attained nonlinearity is reported. Additionally, the mean execution time is shown. The best results obtained for each *n* are shown in boldface. We can see that the cost function C3 attains the best mean nonlinearity in both cases. If we look at the time column, we see that the cost function C3 employs less execution time to achieve a local optimum than the cost function C2.

**Algorithm 1:** Full Hill Climbing (FHC).

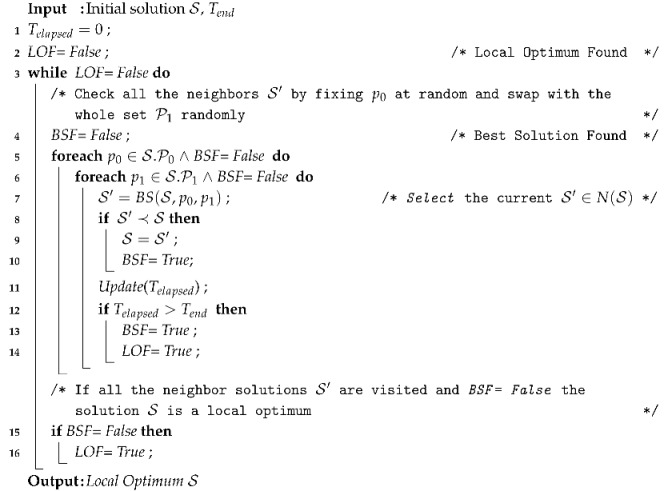



### 4.5. First Improvement Quasi-Tabu Search

FHC with the defined neighborhood is computationally too expensive to be included in a memetic algorithm. Thus, we decided to design an alternative trajectory-based strategy. Tabu Search is a very popular trajectory-based strategy. Two of its main keys are to move to the best neighbor at each iteration and to use a tabu list to avoid cycles. In our case, iterating to get the best neighbor is computationally expensive but applying a tabu list with the aim of reducing the neighborhood seems promising. As a result, a novel method that applies some of the principles of Tabu Search was devised. The strategy is called First Improvement Quasi-Tabu Search (FIQTS) and differently to Tabu Search it moves to the first neighbor that improves the current solution.

Algorithm 2 illustrates the working operation of FIQTS. First, the tabu bit-swapping positions $R_{0}$ and $R_{1}$ are initialized at random (line 2), $R_{0}$ and $R_{1}$ contain positions that are not allowed to bit-swap. Then the non-tabu bit-swapping positions $Q_{0}$ and $Q_{1}$ are initialized (line 3), such that

$$Q_{i}\cup R_{i}=S.P_{i}\;Q_{i}\cap R_{i}=\emptyset \;i\in \left\{0,1\right\}.$$

**Algorithm 2:** First Improvement Quasi-Tabu Search (FIQTS).

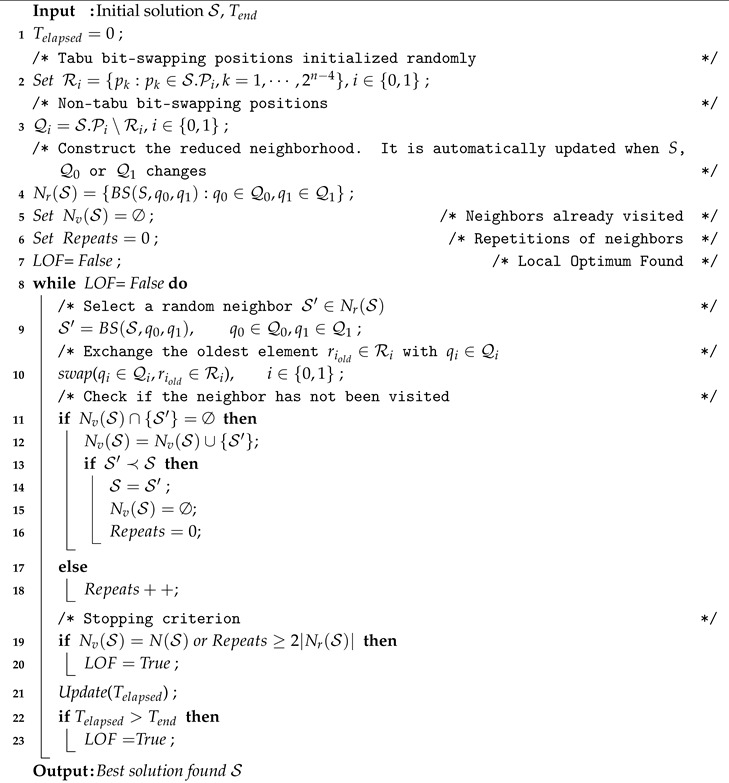



The sets $Q_{0}$ and $Q_{1}$ contain positions such that the decision variables $S[q_{0}]$, $S[q_{1}]$ are set equal to 0, 1 respectively, and that might be bit-swapped. In base of these sets the reduced neighborhood $N_{r}\left(S\right)$ is constructed (line 4). Each time that a neighbor is created by bit-swapping the decision variables in the positions $q_{0}$ and $q_{1}$, such elements are moved to $R_{0}$ and $R_{1}$, respectively (lines 9, 10). Additionally, the elements that have been for a longer time in $R_{0}$ and $R_{1}$ are inserted in $Q_{0}$ and $Q_{1}$. The main principle is to promote the selection of different decision variables at each step when performing the bit-swapping. The stopping criterion is set in such a way that the iterative process ends when all the neighborhood has been visited and no better solution was found or when the random sampling process generates too many neighbors that have already been visited previously (line 19). Another stopping criterion that can be used is time (line 22).

As in the FHC method, FIQTS method was executed with the four cost functions. Table 2 shows the results obtained with FIQTS for *n* = 8, 10. As in the previous case, the cost function C3 attains a better performance. The FIQTS method requires less iterations than the FHC method to reach high-quality solutions, so it is faster to locate local optima.

**Figure 1:**
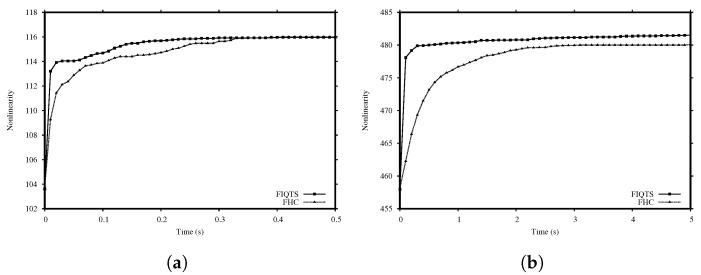
Evolution of the Mean Nonlinearity for FIQTS and FHC. (**a**) 8-variables, (**b**) 10-variables.

According to the results in Table 1 and Table 2, the cost function $C_{3}$ is the most adequate cost function for our optimizers. Thus, the cost function $C_{3}$ is chosen to be used from now on. Moreover, it is noticeable that not only the FIQTS method is slightly better in terms of quality, but it also needs less time. In order to select the method to be integrated in our population-based strategy, it is important to select an inexpensive one with the aim of evolving more generations. In order to decide which method is the most suitable to use in the rest of experiments, we compare the performance of the FHC and FIQTS methods with the cost function $C_{3}$, employing a fixed time of 0.5 and 5.0 s for *n* equal to 8 and 10, respectively. Table 3 shows the results obtained for 50 independent runs for each method. Additionally, Figure 1 shows the evolution of the nonlinearity for the FHC and FIQTS methods with the cost function $C_{3}$. The FIQTS method quickly achieves high nonlinearity, so it is preferable for short-term executions, meaning that this is the method selected to be integrated in our population-based approach.

We would like to note that we also designed trajectory-based strategies based on simulated annealing and iterated local search. However, they were not superior to FIQTS.

## 5. Population-Based Proposals

A very successful way to improve the performance of Evolutionary Algorithms (EAs) is to hybridize with local search or other trajectory-based techniques. In fact, Memetic Algorithms (MAs) [29] that combine genetic algorithms with an individual improvement technique, have been applied successfully to many combinatorial optimization problems. Taking into account the integration between the population-based strategy and the refinement scheme we can classify the MAs in two groups [30]:

- **Lamarckian Memetic Algorithm** (LMA): modifications performed in the individual improvement procedure are written back in every individual representation.
- **Baldwin Memetic Algorithm** (BMA): modifications change the fitness of the individuals without altering its representation.

In our case, we apply a LMA (see Algorithm 3). In order to fully define the LMA the following components have to be defined: *initialization*, *fitness or cost function* for the evaluation step, *mating selection*, *crossover* and *mutation* for the reproduction step, *survivor selection* and *individual improvement*. In the following, we describe each of the different components for our first basic variant. Then, some components that extend them to generate a more effective strategy are presented.

**Algorithm 3:** Lamarckian Memetic Algorithm (LMA).

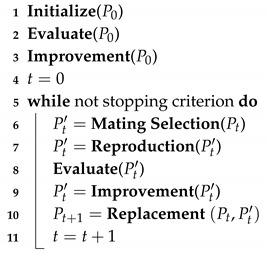



### 5.1. A Lamarckian Memetic Algorithm with a Generational Replacement with Elitism (LMA-GRE)

Our first variant (LMA-GRE) does not consider an explicit control of diversity. In the following the details of its different components are given.

In this first variant, the initial population $P_{0}$ is generated at random. Particularly, *N* balanced BFs are generated following a uniform distribution, that is, each balanced BF is equiprobable. Regarding the evaluation, for each individual the WHT is calculated and then the cost function $C_{3}$ is used to compare individuals. The mating selection is performed with the well-known binary tournament [28]. After the mating selection, the offspring population is built. Two different variation operators that maintain the balance of BFs are applied: *crossover* and *mutation*.

Algorithm 4 describes the working operation of the crossover operator. It is a novel—although quite straightforward—operator. It takes as input two individuals ($I_{j},I_{k}$) and generates two new individuals ($C_{j}$, $C_{k}$). First, it calculates the positions where both individuals contain a one in the truth table (line 3–4). These positions are preserved in the offspring, that is, they are set to one (lines 17–18). Additionally, we calculate the positions that are set to one in only one of the individuals (line 5–6). They are saved in the $R_{1_{j}}$ and $R_{1_{k}}$ sets. Then, until these sets are empty (line 7–16), we select one value randomly for each of the sets. Then, these positions are inherited by the corresponding children (lines 10–11) or exchanged with probability $p_{c}$ (lines 13–14). Note that at each step, the selected positions are removed from the corresponding sets (lines 15–16), and that the remaining positions are set to zero (lines 1–2), meaning that balancedness is maintained.

**Algorithm 4:** Crossover strategy applied in all our optimizers.

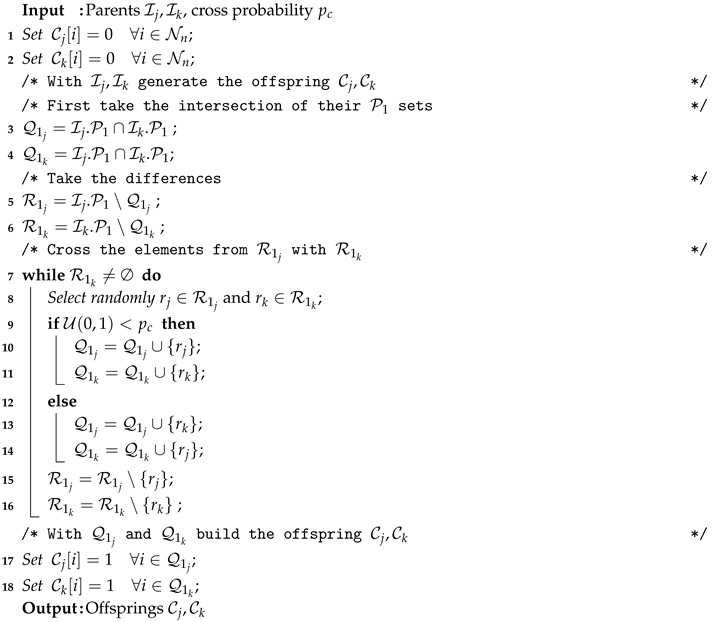



It is important to note that when parents are close to each other, that is, their differences are not large, then the crossover is not very destructive. In particular, if both parents are the same individual no changes are performed. This automatic adaptive behavior is usually appropriate in the design of crossover operators [28].

Regarding the mutation, the applied operator is described in Algorithm 5. The process iterates over all positions set to one and with a given probability ($p_{m}$) it applies the BS operator to exchange this position with a position that contains a zero value. This second position is selected at random.

**Algorithm 5:** Mutation strategy applied in all our optimizers.

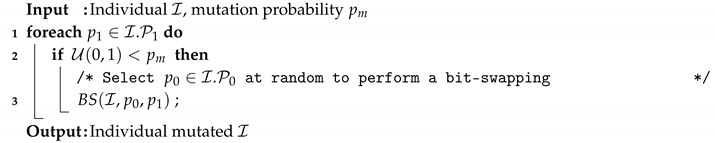



The way in which the mutation and crossover operators are applied is quite standard. Algorithm 6 illustrates the creation of the offspring population by employing the crossover and mutation operators. Note that at each generation *N* offspring are created.

**Algorithm 6:** Reproduction strategy applied in all our optimizers.

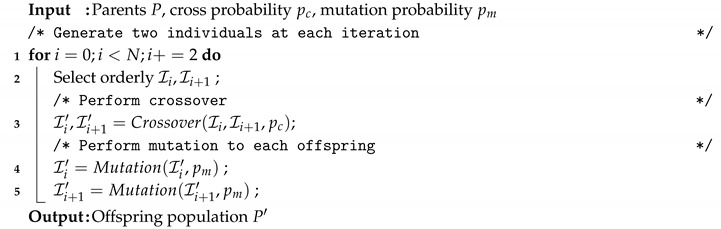



In pure generational schemes, the offspring are the survivors [31]. Since elitism usually contributes to the attainment of high-quality solution some Generational Replacement with Elitism (GRE) variants exist [31]. In our proposal (see Algorithm 7) when the best solution of the previous generation is better than the best offspring, the best solution of the previous generation replaces a randomly selected offspring.

**Algorithm 7:** Generational Replacement with Elitism (GRE) Technique.

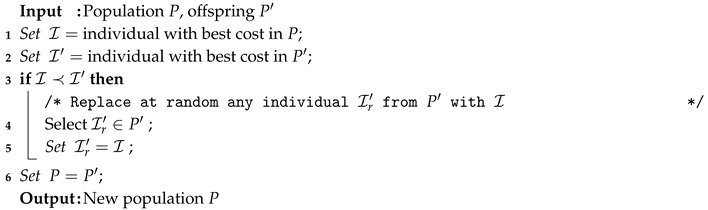



Finally, the intensification procedure must be specified. In LMA-GRE, FIQTS (Algorithm 2) is applied to each offspring. There is just a subtle difference which is the stopping criterion. In this case, FIQTS stops after a certain period of time. The reason for this is that with the aim of evolving a high number of generations, a strict control on the time invested by the intensification procedure is required. Note that LMA-GRE does not incorporate explicit control of diversity, so it is used precisely to validate the advantages of other proposals that incorporate strategies to control the diversity.

### 5.2. LMA with a Replacement Considering Elitism and Dynamic Diversity Control (LMA-REDDC)

The second variant of our optimizer includes a replacement phase that takes diversity into account. Particularly, the replacement phase is similar to the one devised for the Graph Partitioning Problem [10]. This replacement operator (Algorithm 8) operates as follows. It takes as input the population, offspring and a threshold *D* to perform penalties and it selects the members of the next population. First, it selects the individual with the lowest cost (lines 1–6). Then, iteratively it selects the remaining survivors (lines 7–17). Particularly, at each iteration among the non-selected individuals that are not too close to already selected survivors (line 10–11) it selects the one with the lowest cost (lines 12–13). Note that it might happen that all individuals are too close to each other (line 14). In this case, the farthest individual is selected (lines 14–15).

**Algorithm 8:** REDDC Survivor Selection Technique.

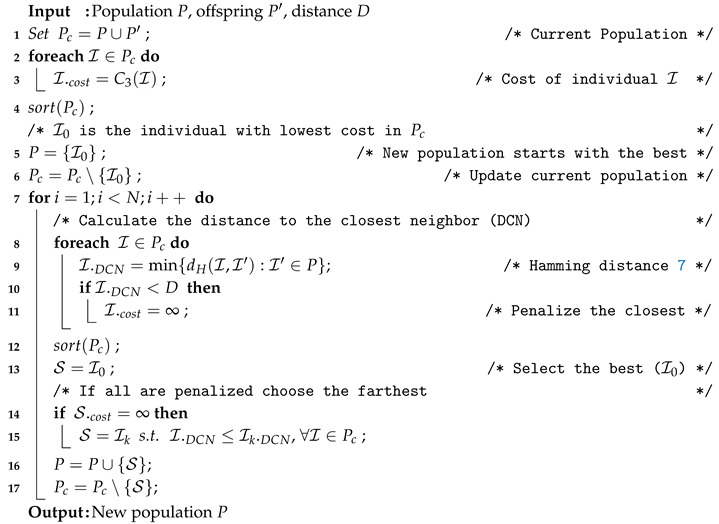



The threshold *D* is quite important. When using a large *D* value, exploration is promoted, whereas small values promote intensification. In order to balance from exploration to exploitation, this threshold is set dynamically following the next equation: $D=D_{0}-\left(D_{0}\right)\times T_{elapsed}/T_{end}$, where $D_{0}$ is a parameter of the optimizer, $T_{elapsed}$ is the elapsed time of the optimization (in seconds), and $T_{end}$ is the stopping criterion (in seconds). This means that at the first generation *D* is set to $D_{0}$ and it decreases linearly so that at the end of the optimization it is set to zero.

### 5.3. A Memetic Algorithm Based on Clusters

Our last variant (MAC-REDDCC) includes two modifications to improve the performance of LMA-REDDC. One of the drawbacks of LMA-REDDC is that in most generations no individual with a distance lower than *D* is accepted (line 10). Usually, when crossover is applied to distant individuals, an exploration step is performed. While maintaining distant individuals to explore different regions is important, using close individuals to better intensify is also important. For this reason, a novel algorithm that tries to explore and intensify during the whole search is proposed.

In order to incorporate this design principle two modifications are incorporated. The most important one is the incorporation of a newly design replacement strategy. The REDDC with Clustering (REDDCC) strategy is explained in Algorithm 9.

**Algorithm 9:** REDDCC Survivor Selection Technique.

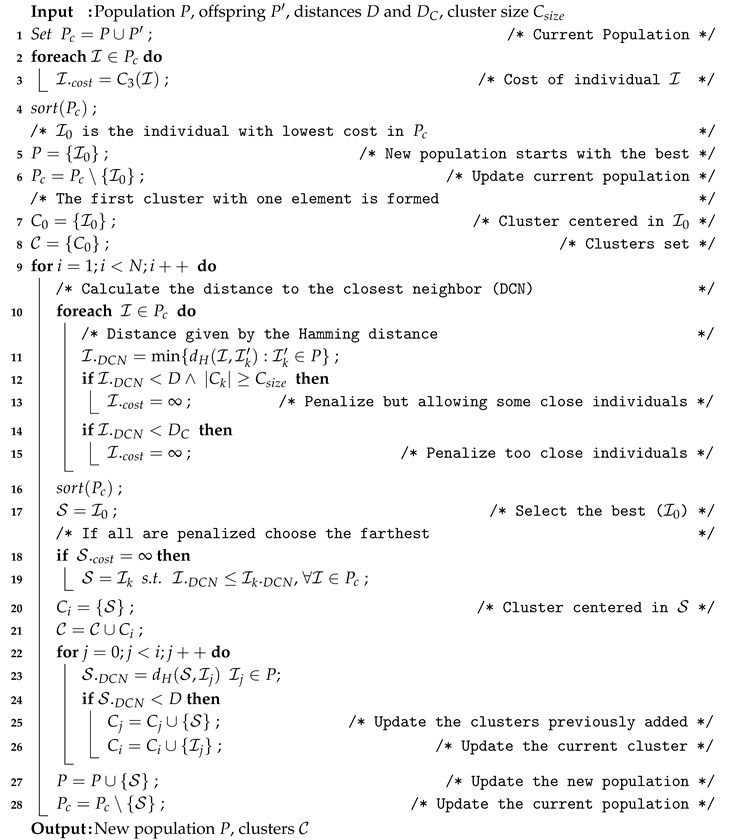



REDDCC alters the replacement strategy by applying a clustering technique that takes into account the stopping criterion. The main difference between the REDDC and the REDDCC strategies is that for each individual the REDDCC strategy allows a certain number of individuals (cluster size $C_{size}$) to be closer than *D*, but farther than $D_{C}<D$ (see lines 12–15), while in the REDDC strategy this is not allowed.

Note that in comparison to previous variants, the general procedure (see Algorithm 10) was also changed. First, since the REDDCC strategy requires $D_{c}$ and *D* as inputs, both values are calculated at each generation (lines 12–15). Some preliminary experiments showed that a larger degree of diversification was only required at initial stages, meaning that *D* and $D_{C}$ could be set to small values after a proportion of the granted execution time ($T_{end}$). Taking this into account, a new parameter, $K_{E}$ is added. This parameter refers to the proportion of time where exploration is promoted (line 6). The updating strategies were designed so that *D* is decreased from $D_{0}$ to $D_{C_{0}}$ and $D_{C}$ is decreased from $D_{C_{0}}$ to 0 after this period of time (lines 13–14). Then, in subsequent generations the values of *D* and $D_{C}$ are not changed (line 15).

**Algorithm 10:** MAC-REDDCC Method.

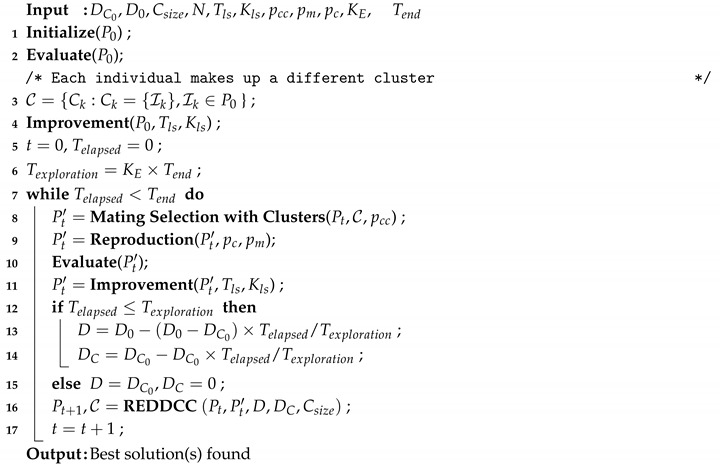



Additionally, a new parameter ($p_{cc}$) is added to facilitate attaining a proper balance between exploration and exploitation. This parameter refers to the probability that two individuals from different clusters are crossed in order to create a pair of offspring. In other cases, individuals that belong to the same cluster are crossed. The new selection process is given in Algorithm 11.

**Algorithm 11:** Mating Selection for MAC-REDDCC.

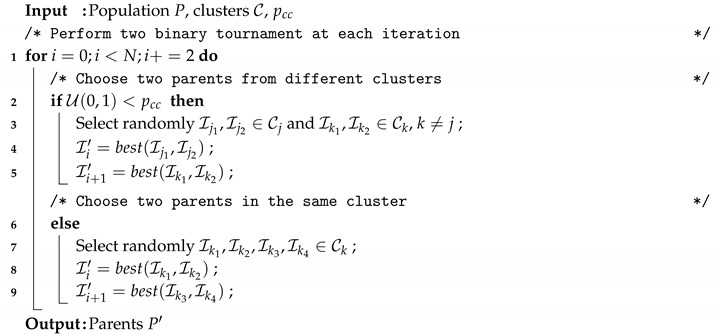



In the case of the improvement phase, the only difference is that we grant more time for local search when the offspring is generated by crossing parents from different clusters. The reason is that in such a case the crossover operator is more disruptive, so it is expected that more time is required to attain a new high-quality solution. Algorithm 12 illustrates the new improvement phase. A new parameter, $K_{ls}$, is added. This parameter is used for setting the time granted to those individuals generated with parents belonging to different clusters (line 3). If the parents of the offspring belong to the same cluster the local search time granted is $T_{ls}$ (line 5).

**Algorithm 12:** Improvement Phase for MAC-REDDCC.

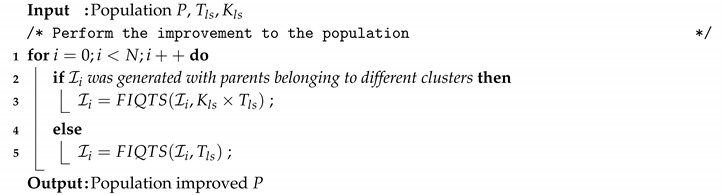



### 5.4. Parameterization Study

One of the inconveniences of the last proposal (MAC-REDDCC) is that several parameters must be set. Particularly these are the following parameters:

1. $D_{C_{0}}$: initial distance threshold used to maintain a proper diversity in each cluster (see Algorithm 10 line 14).
2. $D_{0}$: it is responsible for controlling the degree of diversity maintained in the whole population (see Algorithm 10 line 13).
3. $C_{size}$: indicates the maximum size allowed for each cluster (see Algorithm 9 line 12). Note that since the acceptance of individuals only depends on the information of the closest already selected survivor, some clusters might eventually contain more that $C_{size}$ individuals.
4. *N*: number of individuals in the population and number of offspring generated at each generation (see Algorithm 6 line 1).
5. $T_{ls}$: stopping criterion of the local search procedure for offspring with parents belonging to the same cluster (see Algorithm 12 line 5).
6. $K_{ls}$: if the offspring is generated with parents belonging to different clusters, the local search time applied to this kind of individuals is $T_{ls}\times K_{ls}$ (see Algorithm 12 line 3).
7. $p_{cc}$: indicates the probability to cross individuals belonging to different cluster (see Algorithm 11 line 12).
8. $p_{m}$: probability of performing swaps to mutate the individual (see Algorithm 5 line 2).
9. $p_{c}$: probability of interchanging each gene (see Algorithm 4 line 9).
10. $K_{E}$: indicates the proportion of time with additional promoted exploration (see Algorithm 10 line 6).

In order to properly adjust the parameters, for each parameter 5 different values were tested and since stochastic algorithms are considered, each tested configuration was run 50 times. For each independent run, the stopping criterion was set to 72 h of execution. Due to the limitations of time it is not possible to do an exhaustive search in the parameters space by considering the dependencies between all of them. Particularly, the optimization of each parameter was performed independently from each other. This was done as follows. First, since the problem for 8 variables can be solved easily up to the best-known results for nonlinearity, we focus on the parameters setting for the 10 variable problem. Taking into account some preliminary results, the zero or initial parameter setting was set according to Table 4. With the initial parameter setting we carried out 50 independent runs with the stopping criterion set equal to 72 h. Table 5 shows the results obtained for this experiment. We can see from the results showed in Table 5, that using the initial parameters setting, the algorithm achieves the best results attained by metaheuristics in the 10-variable problem. The mean nonlinearity is high (its mean is 486.96), which indicates that in several of the independent executions it was able to find solutions with nonlinearity equal to 488. The mean nonlinearity will be our main indicator to consider the quality of each parameterization. The seventh column shows the proportion of independent executions that reach this nonlinearity value, which is called the success ratio ($S_{r}$). The initial parameter setting has a success ratio $S_{r}=50\%$, which means that 25 of the 50 independent runs achieve solutions with nonlinearity equal to 488. Since 488 is the maximum nonlinearity that had been obtained ever in a single execution of a metaheuristic, our aim was to find a parameterization with a success ratio close to 100%.

In order to improve the parameterization, each parameter was modified independently maintaining the remaining parameter values as in the best configuration found so far. Parameters were optimized following the order in which they were previously presented. After this parameterization stage we obtained the parameterization shown in Table 6. In this last case the success ratio increased to 100%, meaning that our proposal could generate in every execution the best solution attained up to now by metaheuristics.

### 5.5. Comparison Among Population-Based Metaheuristics

In order to show the benefits of the components included in MAC-REDDCC, it is interesting to compare it against LMA-GRE and LMA-REDDC. All these algorithms were executed using the set of parameters obtained after the parameterization steps. Specifically, each of the three methods were executed 50 independent times with the parameters shown in Table 7.

Table 8 summarizes the attained results. Results obtained by LMA-GRE in the 10-variable case are poor in comparison with the ones obtained with LMA-REDDC and MAC-REDDCC. Thus, the importance of using a scheme based on diversity in order to find proper solutions for the problem of generating BFs with high nonlinearity is clear. When compared to LMA-REDDC, MAC-REDDCC attains slightly better results, so the additional changes performed were useful to increase the robustness and attain a 100% success ratio.

**Figure 2:**
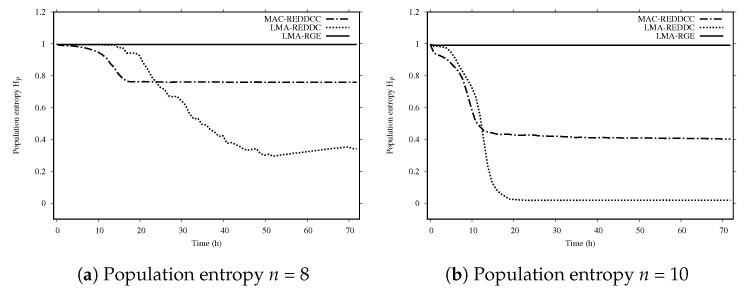
Population entropy *n* = 8.

Figure 2 shows the population entropy for the different evolutionary methods. We can see that the LMA-GRE never converges. LMA-REDDC is the method with the fastest decrease in diversity. The clustering technique attains a larger global entropy with a smaller entropy inside clusters, which is key to the success of MAC-REDDCC.

### 5.6. Hybridization with an Algebraic Technique

Motivated by the related work from Burnett [18] and Izbenko [19], we considered the application of an algebraic procedure to improve our results. Contrary to their work, we decided not to use bent functions, instead we employ the work from Tang [32] to build an initial population of individuals with high nonlinearity. Thus, the only change appears in the *initialization* procedure of the population.

Tang [32] proposes a method capable of constructing balanced BFs of even number of variables with relatively high nonlinearity. Their construction is based on a modification of the Maiorana-McFarland [20] method to construct bent BFs. As a result Tang et al. were able to obtain a large amount of balanced BFs with nonlinearity

(35) $$N_{n}\geq 2^{n-1}-2^{n/2-1}-2^{\left\lceil n/4\right\rceil }.$$

Since in the case of the 8-variable BF problem, we had already obtained the best-known solution, we decided to make experiments with the hybrid method only for the 10-variable BF problem. The initialization strategy allows to generate up to 1310400 BFs with nonlinearity equal to 488. In order to make 50 independent runs and each one with a population size equal to 350, we built 17,500 (50×350=17,500) individuals with nonlinearity equal to 488 with the initialization algorithm [32]. Each population was employed with the three evolutionary methods: MAC-REDDCC, LMA-REDDC and LMA-GRE. The parameter values are the indicated in Table 7. The attained results are shown in Table 9. We can see that the information in Table 9 does not help us to discern which hybrid method is better in comparison with the others because all the methods achieve in all the executions BFs with nonlinearity equal to 492. Note that this is currently the best-known solution and that no meteheuristic had ever reached this value. In order to discern which method is preferable we count the amount of different individuals in the final population that achieve a nonlinearity equal to 492. This comparative is shown in Table 10. From the information in the Table 10, we can see that in MAC-REDDCC and LMA-REDDC every individual in the final population is different and has nonlinearity equal to 492. However, the LMA-GRE is able to maintain only one solution with such nonlinearity. We consider that using the MAC-REDDCC and LMA-REDDC is preferable, because maintaining a diverse population with high quality means that several regions are explored and there is a larger probability to finding better solutions (if they exist). However, when coupling algebraic strategies we are not able to discern between MAC-REDDCC and LMA-REDDC.

**Figure 3:**
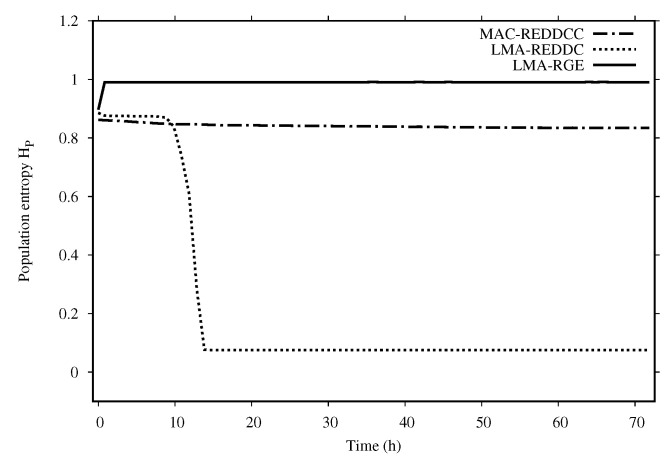
Population entropy for MAC-REDDCC, LMA-REDDC and LMA-GRE when coupled with an algebraic-based initialization.

Figure 3 shows the entropy of the population along the execution time for the three methods tested. As we can see in Figure 3, MAC-REDDCC never decrease its entropy but is able to reach very high quality solutions, so in this sense its behavior seems more adequate to try to reach a solution with a higher nonlinearity.

## 6. Conclusions and Future Work

The problem of generating Cryptographic Boolean Functions (CBFs) with high nonlinearity is an extremely complicated problem. This problem has been addressed with many strategies in the last 30 years. Many heuristic methods have been proposed to generate CBFs with high nonlinearity and the results obtained have been improving over the years. However, the results obtained so far were not as extraordinary as those obtained with algebraic techniques. According to our knowledge, heuristic methods had never been able to generate 10-variable CBFs with nonlinearity equal to 492, but the algebraic constructions do.

In this paper, we work with the hypothesis that a diversity-based metaheuristic can provide a better way of generating CBFs with high nonlinearity, improving further the best current results obtained by other metaheuristic methods and reducing the gap between algebraic and metaheuristic approaches. Based on this, a set of trajectory-based and population-based metaheuristic methods are proposed. Among the proposals, the most novel is a population-based metaheuristic that incorporates explicit diversity management with a clustering technique that allows intensifying and exploring throughout the optimization process, since it forces some members of the population to be distant but some are allowed to be close. This method is called MAC-REDDCC and according to our knowledge, this is the first diversity-aware scheme applied to the generation of CBFs with high nonlinearity.

MAC-REDDCC operates with a set of novel components proposed in this paper. The most important component is the REDDCC strategy, which is responsible for the explicit diversity management. The cost function employed to guide the search is another important component. It takes into account several information from the Walsh Hadamard Transform (WHT), what makes it more suitable to guide the search. The intensification procedure employed to improve the members of the population is another important component. This novel trajectory-based search method is inspired by the Tabu Search algorithm and is called First Improvement Quasi-Tabu Search (FIQTS).

The results obtained with the MAC-REDDCC method improve the best current results reported in the literature by other pure metaheuristic methods, and match the results reported by hybrid metaheuristics with algebraic techniques. As other researchers, we propose a hybridization between the MAC-REDDCC method and a simple algebraic technique. The results obtained with the hybridization match the best results known for 10-variable CBFs, since CBFs with nonlinearity equal to 492 are generated. Thus, MAC-REDDCC is the first metaheuristic method that is able to generate CBFs with that nonlinearity. Our proposal is a single objective mechanism that efficiently solves the problem of creating CBFs for general purposes. This means that it is not difficult to apply it with different fitness or cost functions for different purposes, which opens new lines for future research.

As a future work, the scalability of our proposals should be improved. After performing some initial experiments with 12 variables, we concluded that we need to do some changes—probably in the cost functions and in the local improvement techniques—to be able to deal with 12 or more variables. Particularly, in order to develop a better intensification procedure, it would be interesting to use a neighborhood coupled with the Walsh Hadamard transform. In this way, the improvement process could be faster. In other words, extending the smart hill climbing algorithm to operate with other cost functions seems promising. Additionally, it seems interesting to employ other properties such as the autocorrelation or the algebraic degree inside the cost function. Some authors have obtained improvements with these kinds of transformations, so integrating them with the advances developed in this paper seems promising. Particularly, applying multi-objective optimizers with alternative functions considering more information from the WHT as well as designing ad-hoc operators seems encouraging. Additionally, the applicability of the CBFs generated with our proposals should be further explored. Finally, note that some authors have used specific hardware to speeding up the attainment of high-quality CBFs [33,34]. In order to scale our algorithms, these kinds of hardware as well as supercomputing might be applied.

## Figures and Tables

**Table 1:** Summary of the results attained by FHC applying various cost functions.

n	CF	Min	Max	Mean	Median	$\sigma_{N_{n}}$	$\bar{t}\left(s\right)$
8	$C_{1}$	108	112	110.16	110	1.503	$3.4\times 10^{-2}$
	$C_{2}$	112	116	114.12	114	0.627	$1.6\times 10^{0}$
	$C_{3}$	**114**	**116**	**115.92**	**116**	**0.396**	$2.4\times 10^{-1}$
	$C_{4}$	108	112	111.84	112	0.792	$3.1\times 10^{-2}$
10	$C_{1}$	464	472	469.4	470	1.863	$7.9\times 10^{-1}$
	$C_{2}$	482	484	482.72	482	0.97	$1.8\times 10^{2}$
	$C_{3}$	**480**	**484**	**483.16**	**484**	**1.621**	$1.6\times 10^{1}$
	$C_{4}$	468	480	475.44	476	1.809	$1.9\times 10^{0}$

**Table 2:** Summary of the results attained by FIQTS applying various cost functions.

n	CF	Min	Max	Mean	Median	$\sigma_{N_{n}}$	$\bar{t}\left(s\right)$
8	$C_{1}$	108	112	110.16	110	1.448	$3.8\times 10^{-2}$
	$C_{2}$	112	116	114	114	0.7	$1.1\times 10^{0}$
	$C_{3}$	**114**	**116**	**115.96**	**116**	**0.283**	$1.4\times 10^{-1}$
	$C_{4}$	108	116	112.6	112	1.629	$3.7\times 10^{-2}$
10	$C_{1}$	466	472	470.16	470	1.888	$6.8\times 10^{-1}$
	$C_{2}$	482	484	482.68	482	0.957	$9.4\times 10^{1}$
	$C_{3}$	**480**	**484**	**483.64**	**484**	**1.12**	$1.0\times 10^{1}$
	$C_{4}$	472	480	478.48	480	2.27	$3.4\times 10^{-1}$

**Table 3:** Comparison between FIQTS and FHC with the cost function $C_{3}$ in executions at fixed time.

n	Method	Min	Max	Mean	Median	$\sigma_{N_{n}}$
8	**FIQTS**	**114**	**116**	**115.96**	**116**	**0.283**
	FHC	114	116	115.92	116	0.396
10	**FIQTS**	**480**	**484**	**481.52**	**482**	**1.182**
	FHC	480	482	480.08	480	0.396

**Table 4:** Initial Parameterization used in the Adjustment Phase.

$D_{C_{0}}$	$D_{0}$	$C_{\mathrm{size}}$	*N*	$T_{\mathrm{ls}}$	$K_{\mathrm{ls}}$	$p_{\mathrm{cc}}$	$p_{m}$	$p_{c}$	$K_{E}$
20	100	10	200	0.01	1.0	0.2	0.002	0.5	1.0

**Table 5:** Results attained with the initial parameterization.

n	Min	Max	Mean	Median	$\sigma_{N_{n}}$	$S_{r}$
10	484	488	486.96	487	1.087	50%

**Table 6:** Parameter setting attained after the adjustment procedure.

$D_{C_{0}}$	$D_{0}$	$C_{\mathrm{size}}$	N	$T_{\mathrm{ls}}$	$K_{\mathrm{ls}}$	$p_{\mathrm{cc}}$	$p_{m}$	$p_{c}$	$K_{E}$	**Mean**	$S_{r}$
**14**	**60**	**20**	**350**	**0.08**	**2.0**	**0.2**	**0**	**0.2**	**0.2**	**488**	**100%**

**Table 7:** Parameters involved in MAC-REDDCC, LMA-REDDC and LMA-GRE.

Method	$D_{C_{0}}$	$D_{0}$	$C_{\mathrm{size}}$	*N*	$T_{\mathrm{ls}}$	$K_{\mathrm{ls}}$	$p_{\mathrm{cc}}$	$p_{m}$	$p_{c}$	$K_{E}$
MAC-REDDCC	**14**	**60**	**20**	**350**	**0.08**	**2.0**	**0.2**	**0**	**0.2**	**0.2**
LMA-REDDC	×	**60**	×	**350**	**0.08**	×	×	**0**	**0.2**	**0.2**
LMA-GRE	×	×	×	**350**	**0.08**	×	×	**0**	**0.2**	×

**Table 8:** Comparison between the results attained by the population-based optimizers.

n	Method	Min	Max	Mean	Median	$\sigma_{N_{n}}$
8	MAC-REDDCC	116	116	116	116	0
	LMA-REDDC	116	116	116	116	0
	LMA-GRE	116	116	116	116	0
10	**MAC-REDDCC**	**488**	**488**	**488**	**488**	**0**
	LMA-REDDC	486	488	487.44	488	0.907
	LMA-GRE	482	484	483.92	484	0.396

**Table 9:** Comparison among the results attained by the hybrid methods.

n	Method	Min	Max	Mean	Median	$\sigma_{N_{n}}$
10	MAC-REDDCC	492	492	492	492	0
	LMA-REDDC	492	492	492	492	0
	LMA-GRE	492	492	492	492	0

**Table 10:** Comparison between hybrid methods for the amount of individuals found with nonlinearity equal to 492.

Method	Min	Max	Mean	Median
MAC-REDDCC	350	350	350	350
LMA-REDDC	350	350	350	350
LMA-GRE	1	1	1	1
